# Quantitative cardiovascular magnetic resonance in pregnant women: cross-sectional analysis of physiological parameters throughout pregnancy and the impact of the supine position

**DOI:** 10.1186/1532-429X-13-31

**Published:** 2011-06-27

**Authors:** Alexia Rossi, Jerome Cornette, Mark R Johnson, Yusuf Karamermer, Tirza Springeling, Petra Opic, Adriaan Moelker, Gabriel P Krestin, Eric Steegers, Jolien Roos-Hesselink, Robert-Jan M van Geuns

**Affiliations:** 1 Department of Cardiology, Erasmus University Medical Center, 's Gravendijkwal 230, 3015CE Rotterdam, The Netherlands; 2 Department of Radiology, Erasmus University Medical Center, 's Gravendijkwal 230, 3015CE Rotterdam, The Netherlands; 3 Department of Obstetrics and Gynaecology, Division of Obstetrics and Prenatal Medicine, Erasmus University Medical Center, 's Gravendijkwal 230, 3015CE Rotterdam, the Netherlands; 4 Department of Obstetrics and Gynaecology, Imperial College of Medicine, Chelsea and Westminster Hospital, London, UK

## Abstract

**Background:**

There are physiological reasons for the effects of positioning on hemodynamic variables and cardiac dimensions related to altered intra-abdominal and intra-thoracic pressures. This problem is especially evident in pregnant women due to the additional aorto-caval compression by the enlarged uterus. The purpose of this study was to investigate the effect of postural changes on cardiac dimensions and function during mid and late pregnancy using cardiovascular magnetic resonance (CMR).

**Methods:**

Healthy non-pregnant women, pregnant women at 20^th ^week of gestation and at 32^nd ^week of gestation without history of cardiac disease were recruited to the study and underwent CMR in supine and left lateral positions. Cardiac hemodynamic parameters and dimensions were measured and compared between both positions.

**Results:**

Five non-pregnant women, 6 healthy pregnant women at mid pregnancy and 8 healthy pregnant women at late pregnancy were enrolled in the study. In the group of non-pregnant women left ventricular (LV) cardiac output (CO) significantly decreased by 9% (p = 0.043) and right ventricular (RV) end-diastolic volume (EDV) significantly increased by 5% (p = 0.043) from the supine to the left lateral position. During mid pregnancy LV ejection fraction (EF), stroke volume (SV), left atrium lateral diameter and left atrial supero-inferior diameter increased significantly from the supine position to the left lateral position: 8%, 27%, 5% and 11%, respectively (p < 0.05). RV EDV, SV and right atrium supero-inferior diameter significantly increased from the supine to the left lateral position: 25%, 31% and 13% (p < 0.05), respectively. During late pregnancy a significant increment of LV EF, EDV, SV and CO was observed in the left lateral position: 11%, 21%, 35% and 24% (p < 0.05), respectively. Left atrial diameters were significantly larger in the left lateral position compared to the supine position (p < 0.05). RV CO was significantly increased in the left lateral position compared to the supine position (p < 0.05).

**Conclusions:**

During pregnancy positional changes affect significantly cardiac hemodynamic parameters and dimensions. Pregnant women who need serial studies by CMR should be imaged in a consistent position. From as early as 20 weeks the left lateral position should be preferred on the supine position because it positively affects venous return, SV and CO.

## Background

Increasing numbers of women with pre-existing heart disease are reaching childbearing age and are deciding to become pregnant [1]. Pregnancy induces marked physiological changes in cardiac parameters, with a 30-50% increase in cardiac output, through an increase both in stroke volume and heart rate [2]. While usually well tolerated in healthy pregnant women, these changes can induce adverse effect in women with pre-existing heart disease on both right and left-sided lesions [3,4]. Therefore, heart function should be closely monitored during pregnancy in these patients. Echocardiography has been used for many years but cardiovascular magnetic resonance (CMR) is more reliable in the context of congenital heart disease [5]. To date, most data have been derived using echocardiography with the patients in lateral position [6], while CMR is usually performed in supine position. As aortocaval compression is important in advanced pregnancy [7,8] data of both techniques can not be compared. Many women with complex cardiac conditions will require CMR during pregnancy, however there is relatively little data regarding both the use of CMR during pregnancy and of the impact of supine and lateral positions on cardiac parameters. The purpose of this study was to investigate the impact of maternal position on cardiac parameters derived from CMR during 2^nd ^and 3^rd ^trimesters of pregnancy in normal women.

## Methods

### Patient selection

Healthy non-pregnant women, pregnant women at 20^th ^week of gestation and at 32^nd ^week of gestation with no history of cardiac disease were recruited to the study between June 2009 and January 2010. Study participants underwent CMR in supine and left lateral positions. Exclusion criteria were the common contraindications for CMR studies (pacemaker, cochlea implants and claustrophobia). The study was approved by the institutional review board and each subject gave informed consent.

### CMR protocol

CMR was performed using a 1.5T scanner (Signa CV/*I*, GE Medical System, Milwaukee, WI). Firstly the patient was placed in the supine position and entered feet first into the magnet. A dedicated cardiac 8 channels coil was placed on the thorax of the subject and used for the acquisition of the images. CMR cines were obtained using a breath-holding ECG triggered balanced steady state free precession sequence. Imaging parameters were as follows: FOV 36-40 × 28-32 cm; matrix 224 × 196; TR: 3.4 milliseconds; TE: 1.5 milliseconds; flip angle 45 degrees; 12 views per segment. Slice thickness was 8 mm with a gap of 2 mm. These parameters resulted in a temporal resolution per image of 41 milliseconds. At first, three rapid surveys were obtained for the determination of the cardiac position and orientation; two- and four-chamber cine MR images were then obtained. The series of short axis (SA) images were obtained from the reference images provided by the two- and four- chamber end-diastolic images at the end of expiration. Approximately 10 to 12 slices were acquired to cover the entire length of the heart. Directly after the first CMR study, the subject was repositioned on the left lateral side position for the second examination. The acquisition of images was performed by the same operator.

### Image analysis

**Figure 1:**
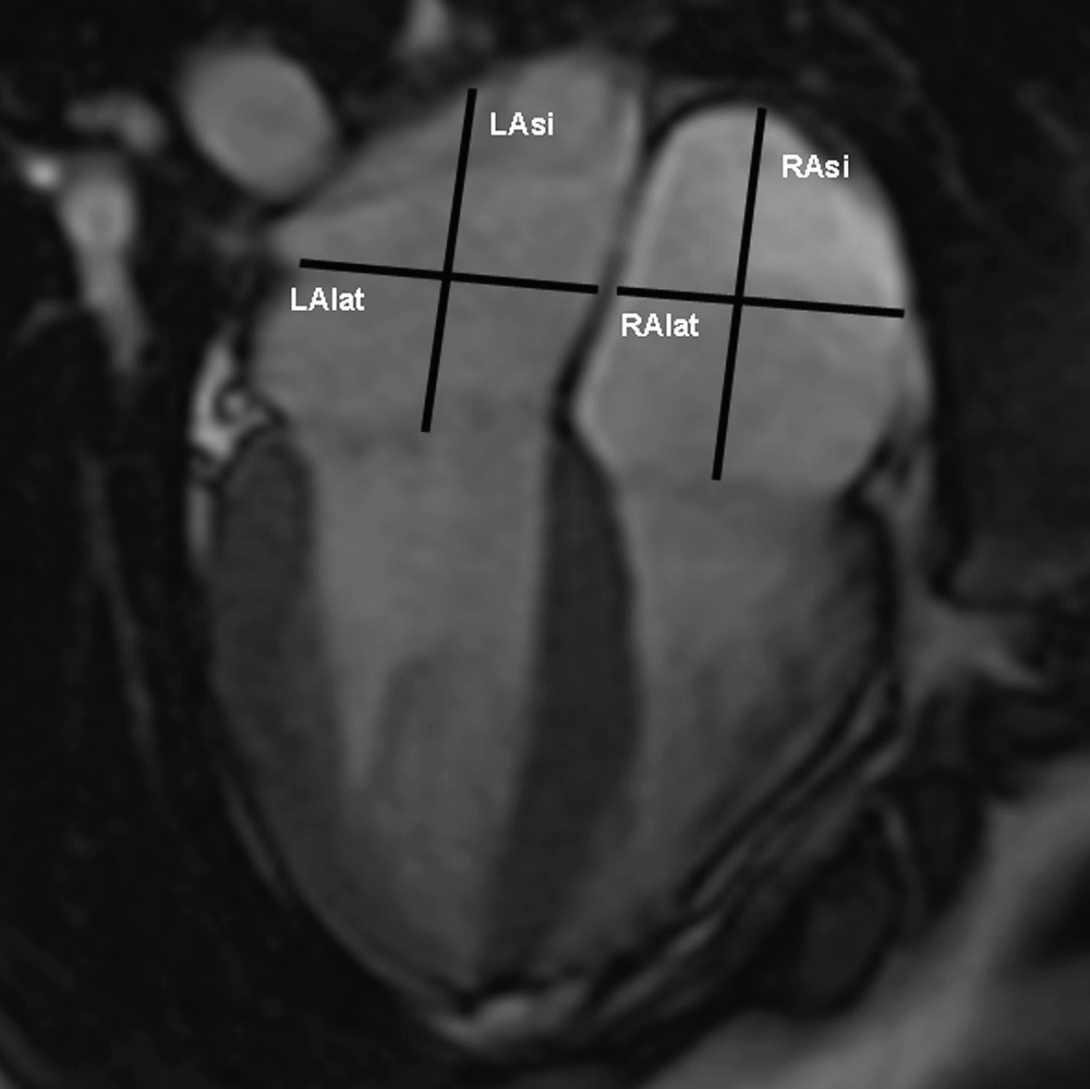
**Cardiac dimensions: four-chamber end-systolic view**. LAlat: left atrium lateral diameter; LAsi: left atrium supero-inferior diameter; RAlat: right atrium lateral diameter: RAsi: right atrium supero-inferior diameter.

All the studies were analysed on a remote workstation using the CAAS-MRV (version 3.2; Pie Medical Imaging, Maastricht, The Netherlands). Left end-diastolic volume (EDV) and end-systolic volume (ESV) were calculated using a combination of classic SA and long-axis images. The long-axis view was used to limit the extend of volumes at the base and at the apex of the heart. The Simpson rule was used to calculate volumes based on the SA images where the first basal and the last apical were only partially included relating to the area outlined on two- and four- chamber images. More details about this approach have been previously reported [9]. The papillary muscles were considered as being part of the blood pool shortening the analysis time without compromising the accuracy of LV volumes compared to standard short-axis technique [10]. Ejection fraction (EF) was calculated as (EDV - ESV)/EDV. Cardiac output (CO) was calculated from stroke volume (SV) and heart rate (HR). Left atrium volume (LAvol) was measured using a combination of the two- and four chamber views in the diastolic phase of the atria. Lateral (LAlat) and supero-inferior (LAsi) left atrial diameters were measured on a four-chamber view during the phase of cardiac cycle with the largest left atrium (Figure 1). The lateral diameter was taken from the perpendicular constructed from the midpoint of the LAsi diameter extending to the atrial borders. The LAsi dimension corresponds to a line bisecting the left atrium and extending from the midpoint of the mitral annulus to the midpoint of the superior left atrium.

Right EF, EDV, ESV, SV and CO were calculated using cine images acquired in SA view, parallel to the tricuspid valve annulus and applying the same methods of the left ventricular hemodynamic measurements without long-axis corrections. In addition, lateral (RAlat) and supero-inferior (RAsi) right atrium diameters were taken during the phase of the cardiac cycle with the largest right atrium, on a four-chamber view (Figure 1). The RAlat diameter corresponds to the line extending from the atrial borders and perpendicular to the RAsi diameter. The RAsi diameter is the line from the midpoint of the tricuspid valve to the midpoint of the superior right atrium.

Due to the low inter-observer variability reported in a previous study [11] image analysis was performed by one operator with 3 years-experience in CMR. The operator was blinded to the same patient at the other position. CMR analyses were performed in a random order at different days.

### Analysis by gestational week

The patients were categorized into 3 groups according to gestational age. The first group consists of non-pregnant controls, the second group of women in the 20^th ^gestational week and the third group of women in the 32^nd ^gestational week.

### Statistical analysis

All analyses were done using SPSS 15 (SPSS Inc.) software. Parametric data were reported as mean ± standard deviation. For each gestational group mean values of HR, EDV, ESV, EF, SV, CO, left and right atrium diameters of supine and left lateral positions were tested for significance, using Wilcoxon's two sample test. A p-value < 0.05 was considered significant. The percentage of change in the measure of left ventricle (LV) and right ventricle (RV) parameters (X) and left atrium (LA) and right atrium (RA) parameters (X) between supine and left lateral position was calculated using the following formula:

## Results

**Figure 2:**
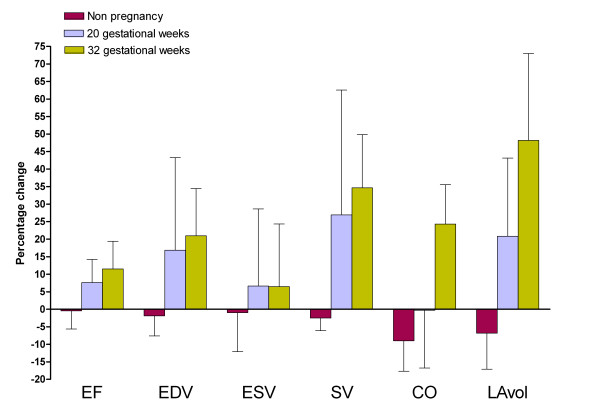
**Percentage differences of hemodynamic parameters of left side of the heart.** EF (ejection fraction: %), EDV (end-diastolic volume: ml), ESV (end-systolic volume: ml), SV (stroke volume: ml), CO (cardiac output: L/min), LAvol (left atrium volume: ml) Percentage difference from supine to left lateral position is calculated with the following formula: X (%) = [(X_lateral _- X_supine_)/(X_supine_)] × 100 where X is a cardiac parameter.

**Figure 3:**
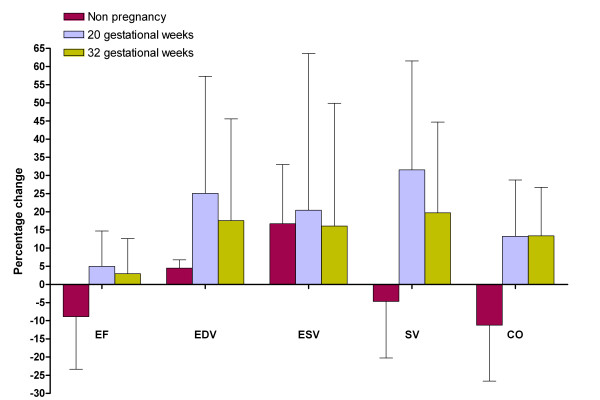
**Percentage differences of hemodynamic parameters of right side of the heart.** EF (ejection fraction: %), EDV (end-diastolic volume: ml), ESV (end-systolic volume: ml), SV (stroke volume: ml), CO (cardiac output: L/min), LAvol (left atrium volume: ml) Percentage difference from supine to left lateral position is calculated with the following formula: X (%) = [(X_lateral _- X_supine_)/(X_supine_)] × 100 where X is a cardiac parameter.

A total of 14 healthy women with singleton pregnancies (30.3 ± 5.2 years) were included in the study. Five non-pregnant women (29.4 ± 5.7 years) were recruited as controls. The time interval between the examinations in supine and left lateral position ranged between 8 and 12 minutes. Table 1 gives the data regarding hemodynamic parameters and cardiac dimensions as related to gestational week and maternal posture. Percentage differences of cardiac volumes between supine and left lateral position are graphically reported in Figure 2 and 3 and will be reported in more detail below.

**Table 1:** Influence of position related to gestational weeks.

	Pre pregnancy (N = 5)					T20 (N = 6)					T32 (N = 8)				
	**Supine position** **Mean (SD)**		**Left lateral position** **Mean (SD)**		**p-value**	**Supine position** **Mean (SD)**		**Left lateral position** **Mean (SD)**		**p-value**	**Supine position** **Mean (SD)**		**Left lateral position** **Mean (SD)**		**p-value**

**LEFT ATRIUM AND LEFT VENTRICLE**															

**HR**	78.2	(12.7)	73.0	(12.1)	0.180	80.5	(11.3)	72.3	(5.3)	0.104	80.8	(15.7)	75.2	(10.6)	0.237

**EF (%)**	56.2	(3.5)	55.9	(4.3)	0.686	53.8	(4.4)	57.8	(4.6)	0.046	51.8	(7.2)	57.7	(8.0)	0.012
**EDV (ml)**	156.5	(23.3)	153.5	(24.7)	0.686	140.8	(37.1)	157.4	(20.7)	0.115	138.4	(22.1)	166.1	(25.6)	0.012
**ESV (ml)**	68.6	(12.7)	67.8	(14.1)	0.893	64.6	(17.9)	66.5	(11.8)	0.917	67.1	(16.4)	71.4	(22.3)	0.484
**SV (ml)**	87.8	(12.9)	85.7	(13.4)	0.223	76.0	(21.1)	90.9	(13.4)	0.028	71.2	(12.2)	94.8	(13.1)	0.012
**CO (L/min)**	6.8	(0.8)	6.2	(1.2)	0.043	6.5	(1.1)	6.5	(1.6)	0.917	5.6	(0.7)	6.9	(0.9)	0.012
**LA vol (ml)**	65.5	(13.3)	57.1	(15.8)	0.068	51.8	(12.7)	60.8	(10.3)	0.028	49.0	(20.9)	68.6	(15.7)	0.012
**LA lat (mm)**	39.7	(2.8)	37.8	(3.3)	0.080	35.0	(3.9)	36.7	(3.7)	0.028	35.4	(2.4)	40.7	(3.7)	0.012
**LA si (mm)**	45.1	(6.7)	43.7	(3.4)	0.500	40.3	(3.9)	44.9	(3.2)	0.027	39.7	(6.3)	44.7	(8.2)	0.025
**RIGHT ATRIUM AND RIGHT VENTRICLE**															
**EF (%)**	51.5	(5.5)	46.4	(4.0)	0.225	50.5	(3.4)	52.9	(4.7)	0.249	53.2	(6.8)	54.6	(6.6)	0.575
**EDV (ml)**	161.0	(24.8)	168.6	(29.2)	0.043	132.0	(34.6)	156.1	(22.3)	0.028	138.8	(37.3)	155.9	(22.6)	0.161
**ESV (ml)**	78.7	(18.8)	90.5	(17.6)	0.080	65.1	(16.5)	73.1	(8.9)	0.463	65.7	(22.6)	71.8	(18.7)	0.484
**SV (ml)**	82.3	(10.7)	78.1	(14.1)	0.345	66.9	(19.3)	84.1	(16.9)	0.028	73.1	(17.2)	84.1	(7.9)	0.050
**CO (L/min)**	6.4	(1.0)	5.6	(1.1)	0.138	5.3	(1.5)	5.9	(1.8)	0.075	5.6	(0.6)	6.3	(0.7)	0.025
**RA lat (mm)**	34.4	(5.0)	33.5	(5.2)	0.345	32.4	(4.0)	33.2	(5.2)	0.500	33.9	(6.2)	36.2	(7.1)	0.484
**RA si (mm)**	47.5	(2.8)	45.9	(4.4)	0.345	40.4	(4.4)	45.1	(5.6)	0.042	44.5	(6.0)	45.2	(7.8)	0.889

T20: 20^th ^gestational week; T32: 32^nd ^gestational week.

EF (ejection fraction), EDV (end-diastolic volume), ESV (end-systolic volume), SV (stroke volume), CO (cardiac output), LAvol (left atrium volume), LAlat (left atrium lateral diameter), LAsi (left atrium supero-inferior diameter), RAlat (right atrium lateral diameter), RAsi (right atrium supero-inferior diameter)

### Pre pregnancy

HR did show a slight although not significantly decrease between supine and left lateral position: 78 ± 12 versus 73 ± 12. Left CO significantly decreased by 9% (p = 0.043) and right EDV significantly increased by 5% (p = 0.043). There were no other significant changes of hemodynamic parameters and cardiac dimensions between the two recumbent positions.

### 20 gestational weeks

Six pregnant women were in the 20^th ^gestational week. HR was 80 ± 11 bpm in the supine position and 72 ± 5 in the left lateral position (p = 0.15). A significant increment of EF and SV of the left ventricle was observed between the supine and the left lateral position: 8% (p = 0.046) and 27% (p = 0.028), respectively. Left atrial dimensions increased significantly between the supine and the left lateral position by 5% for LAlat (p = 0.028) and by 11% for LAsi (p = 0.027). Regarding the right side of the heart, EDV increased by 25% (0.028) and SV increased by 31% (0.028) between the supine and the left lateral position. RAsi significantly increased by 13% (p = 0.042) between the supine and the left lateral position.

### 32 gestational weeks

Eight pregnant women were in the 32^nd ^gestational week. HR did not significantly change between the supine and the left lateral position: 81 ± 16 versus 75 ± 8 bpm (p = 0.237). A significant increment of EF, EDV, SV and CO was observed between the supine and the left lateral position: 11% (p = 0.012), 21% (p = 0.012), 35% (p = 0.012) and 24% (p = 0.012), respectively. Left atrial dimensions increased significantly between the supine and the left lateral position by 15% for LAlat (p = 0.012) and by 13% for LAsi (p = 0.025). No significant changes of right hemodynamic parameters and dimensions were observed between the two recumbent positions with the only exception of CO (p = 0.025).

### Impact of gestational age

A progressive increase of percentage changes of hemodynamic parameters and cardiac dimensions of the left side of the heart was found throughout gestation. The only exception was the percentage change of ESV which substantially did not change between 20 and 32 weeks of gestation. During late pregnancy left ventricle CO significantly increased between supine and left lateral position; the percentage of increment at 32 weeks was 24%. This was associated to an increase of 21% of left ventricle EDV. A significant increase in LA dimensions was also found. The difference between the two recumbent positions was less clear at 20^th ^gestational week with an increase of left ventricle EDV and LA dimensions but with no clear impact on CO. For the right side of the heart we observed a similar trend of increment of ventricular and atrial dimensions at 20 weeks but no further increase during late pregnancy.

## Discussion

This study investigated how the supine and the left lateral positions during CMR affects heart rate, cardiac volumes and dimensions at different gestational ages. To our knowledge this is the first study investigating the effect of two recumbent positions on cardiovascular hemodynamic measurements and changes in cardiac dimensions during pregnancy using CMR. The data show a clear difference between the two positions, which become more marked as pregnancy advances but are significant from as early as 20 weeks. There were minimal changes in the non-pregnant subjects. The existing data from non-pregnant humans with regards to the effect of different positions on hemodynamic parameters are limited and sometimes conflicting. Some report that cardiac output is higher in the supine position compared to the right or left lateral position [12,13], while others have shown that cardiac output is higher in the left lateral position than in the supine position [14] and still others that there is no difference in either position [15,16]. In our series, we found that there was no significant effect on hemodynamic parameters and cardiac dimensions of moving from the supine to the left lateral position in non-pregnant women. However, we did observe a non significant decline in both heart rate and stroke volume leading to a borderline significant reduction in cardiac output.

Pregnancy itself is a circulatory burden with a significant impact on the cardiovascular system. Cardiac output increases 30-50% above pre-pregnancy levels. In addition, when a pregnant woman lies flat on her back, the gravid uterus partially compresses the inferior vena cava with the consequent reduction of venous filling load and cardiac output [2,17]. From our data it appears that the increase of left atrial volume accounts for the majority in increase in stroke volume. As such it seems that the relief of caval obstruction (preload) is far more important that the relief of aortic compression (afterload) for the increase in cardiac output. In our series the significant increase in LA dimensions and the trend to increase EDV in the left lateral position suggest an increased venous return in this position which is already present at 20 gestational weeks. The effect of the gravid uterus compressing the abdominal vessels might be enforced by the increase in plasma volume even as early as 20 weeks of pregnancy [2]. An increase of right atrial pressure [18] and left and right ventricular peak systolic and end-diastolic pressures [19] in the left lateral position can also help to explain the increased venous return. It is of interest to observe that the heart rate was higher in the supine position compensating for the fall in stroke volume in this position [16] in an attempt to recover cardiac output. This could be partly explained by the fact that during mid-late pregnancy the suppression of cardiac vagal activity and the enhancement of cardiac sympathetic activity are greater in the supine position [20] than in the lateral position. In this series left ventricle EDV, EF, CO and SV increase significantly from the supine to left lateral position. These findings are easily explained: the change from the supine to the lateral position relieves the compression on the vena cava from the gravid uterus. The increasing venous return leads to an increased SV and so CO. Ueland et al [16] demonstrated that a change in position from the supine to the left lateral side produced a rise in CO by 8% at 20 to 24 weeks gestation, 13.6% at 28 to 32 weeks gestation and 28.5% at term in a group of eleven healthy pregnant women. We observed that the SV increased by approximately 27% at 20 weeks gestation. At 32 weeks gestation SV increased significantly by 35%. In our series turning to the left lateral position we observed an increment of the SV of the right ventricle which is more evident in the mid than in the late pregnancy. More studies are needed to better explain the consistency of this finding.

Some concerns may develop regarding the use of CMR in pregnant patients. Most studies evaluating MR safety during pregnancy do not show ill effects on the fetus [21-23]. It is anyway good practice to avoid MR studies during the first trimester of pregnancy although it can be used if clinical indicated. All our patients were studied during the second or third trimester of pregnancy.

Several limitations of this study should be highlighted. First, our results should be tested in a larger sample of women including women with cardiac disease. Indeed the normal physiological respond to pregnancy could be different in patients with congenital or acquired cardiovascular diseases. In addition, the small sample size may justify the large standard deviation in our series. Second, because the interval between each MR acquisition was between 8 and 12 minutes it is possible that cardiac parameters had not returned to baseline. Additional studies using more time points and also investigating the reverse change from lateral to supine could clarify this interesting subject. Third, this study is a cross-sectional study. The effects of the position on cardiac hemodynamic should preferably be studied in a prospective-longitudinal study investigating the same population at different gestational times.

## Conclusion

In the non-pregnant state, turning from supine to left lateral position has minimal effect on cardiac parameters. During pregnancy, from as early as 20 weeks, turning to the left lateral position has positive effects on cardiac hemodynamics inducing a significant increase of venous return, SV and CO. Pregnant women requiring CMR should be studied in a consistent position for serial studies and the left lateral position is preferred from early pregnancy onwards, also to limit uteroplacental hypoperfusion.

## List of abbreviations

CMR: cardiovascular magnetic resonance; SA: short-axis; EDV: end-diastolic volume; ESV: end-systolic volume; EF: ejection fraction; SV: stroke volume; CO: cardiac output; LV: left ventricle; RV: right ventricle; LA: left atrium; RA: right atrium; LAlat: lateral left atrium diameter; LAsi: supero-inferior left atrium diameter; RAlat: lateral right atrium diameter; RAsi: supero-inferior right atrium diameter

## Competing interests

The authors declare that they have no competing interests.

## Authors' contributions

The contributions of each author to this manuscript are as follows; AR was involved in the acquisition, analysis and interpretation of the data and drafting of the manuscript. YK, TS and PO were involved in the conception and acquisition of the data. JC, MRJ, AM, GPK, ES were involved in revising the manuscript critically for important intellectual content. JRH was involved in the design of the study and revising the manuscript. RJG was responsible for the conception and design of the study, interpretation of the data and drafting the manuscript. All authors have read and approved the final manuscript.
